# Case Report: Successful treatment of pulmonary lymphomatoid granulomatosis with a PD-1 inhibitor-based regimen

**DOI:** 10.3389/fonc.2025.1700813

**Published:** 2026-01-09

**Authors:** Xiaoyan Liu, Qi Gao, Feifei Wu, Junli Jia, Li Cao, Qingfeng Yu, Dandan Zhang, Wugan Zhao, Jie Ma

**Affiliations:** 1 Department of Hematology, The First Affiliated Hospital of Zhengzhou University, Zhengzhou, Henan, China; 2 Department of Respiratory, The First Affiliated Hospital of Zhengzhou University, Zhengzhou, Henan, China; 3 Department of Hematology, The First Affiliated Hospital of Nanyang Medical College, Nanyang, Henan, China; 4 Department of Pathology, The First Affiliated Hospital of Zhengzhou University, Zhengzhou, Henan, China

**Keywords:** PD-1 inhibitor, pulmonary lymphomatoid granulomatosis, rituximab-based regimens, survival time, treatment strategy

## Abstract

Pulmonary lymphomatoid granulomatosis (PLG) is a rare and aggressively progressive tumor characterized by atypical clinical manifestations and pathological features. This condition is highly prone to misdiagnosis and underdiagnosis. The absence of standardized treatment regimens has resulted in diverse recommendations in the literature, predominantly favoring rituximab-based therapies. However, the prognosis for these patients remains poor, with a median survival time of only 14 months. Recently, we encountered a case of PLG exhibiting programmed cell death-1 (PD-1), PD-L1, and p53 expression. The patient was treated with a PD-1 inhibitor-based regimen. Remarkably, the patient achieved an overall survival (OS) of 52 months at the most recent follow-up, without disease progression. This case stands as a notable observation, particularly because the utilization of a PD-1 inhibitor-based regimen has not been previously reported for PLG treatment. We hope this case will contribute significantly to enhancing physicians’ understanding of PLG and provide a new potential treatment strategy.

## Introduction

Lymphomatoid granulomatosis (LYG) is a rare B-cell clonal lymphoproliferative disorder, frequently associated with Epstein–Barr virus (EBV) infection, according to current studies (1). Initially described in 1972 by Liebow et al. (2), LYG presents clinical manifestations similar to granulomatosis with polyangiitis (GPA), with histological features resembling lymphoma. LYG manifests as a multisystem disorder, potentially affecting the kidneys (45%), skin (25%–50%), and central nervous system (25%–50%). Notably, the lung is the predominant organ involved, with an incidence exceeding 90%, classifying it as a type of primary pulmonary B-cell lymphoma (PP-BCL) (3). Pulmonary lymphomatoid granulomatosis (PLG) is an aggressive disease that has been reported to achieve long-term remission without treatment in 14% to 27% of cases. Conversely, it proves fatal in 63.5% of patients, with a reported median survival time of 14 months (4). The limited understanding of the pathogenesis, the scarcity of reported cases, and the absence of an established treatment protocol for LYG have contributed to its poor prognosis (4). We recently diagnosed a case presenting with programmed cell death-1 (PD-1)/PD-L1 and p53 expression and treated with a PD-1 inhibitor-based regimen, resulting in an overall survival of approximately 52 months. To our knowledge, LYG treated with a PD-1 inhibitor-based regimen has not been previously described. We herein report the case as follows.

## Case report

**Figure 1:**
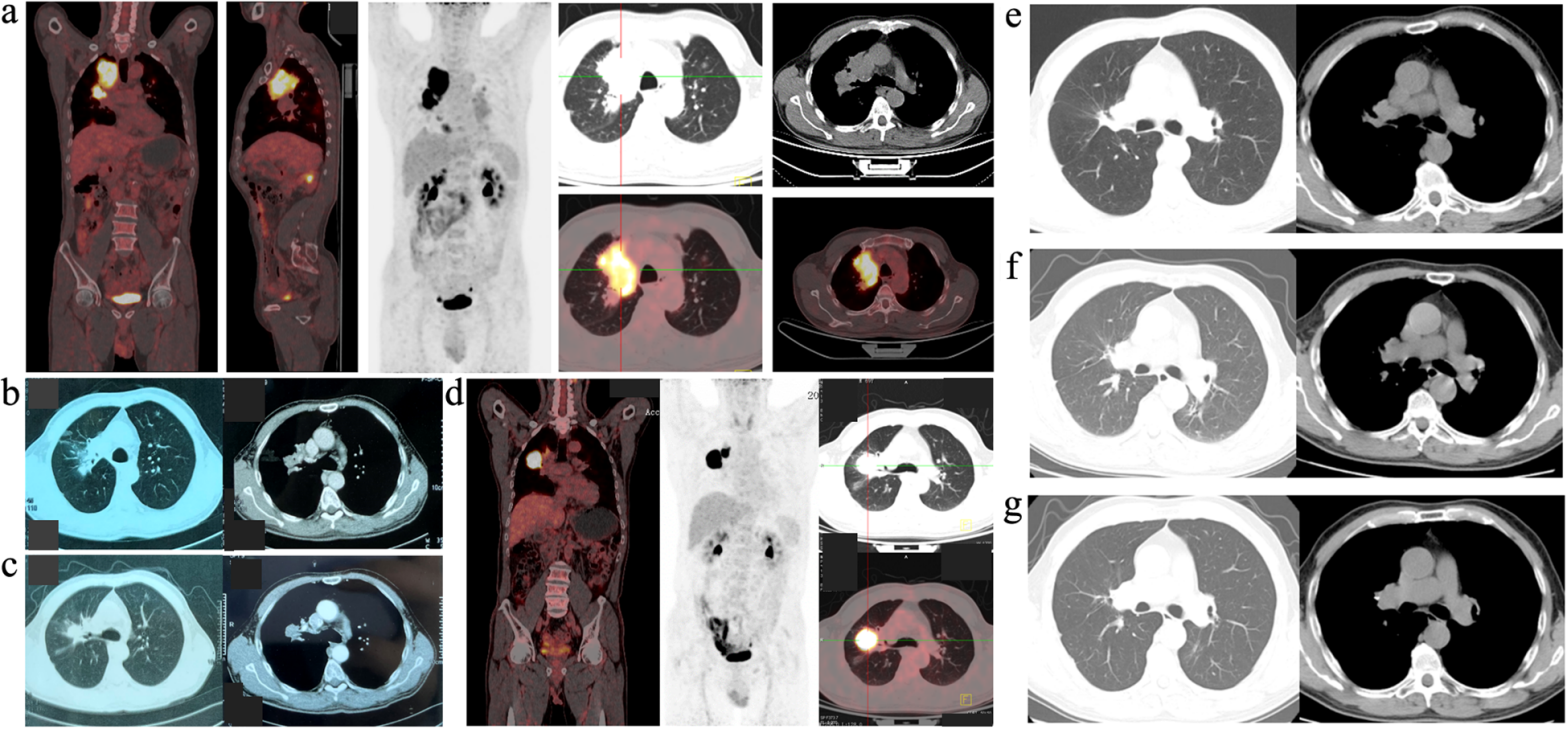
**(a)** PET-CT of the patient at diagnosis. **(b)** Enhanced CT after the patients had two cycles of treatment. **(c)** Enhanced CT after the patients had four cycles of treatment. **(d)** PET-CT after he had six cycles of treatment. The metabolically active mass was still present, and the SUVmax was about 39.1 and the maximum diameter was 3.5cm plus 3.9cm. **(e)** Enhanced CT of the hilar lesions after the patient had eight cycles of chemotherapy. **(f)** Enhanced CT of the hilar lesions after the patient had twelve cycles of chemotherapy. **(g)** Enhanced CT of the patient in the last follow-up time (March 3, 2020).

A 69-year-old male patient was initially admitted to the thoracic surgery department of our hospital in May 2021 due to a mass in the right upper lung that had persisted for over 8 months. He reported no symptoms such as fever, cough, expectoration, shortness of breath, night sweats, or weight loss, and had a history of good health. On physical examination, no abnormalities were detected in either lung, and there were no findings of superficial lymphadenophathy and hepatosplenomegaly. Routine blood tests and lung cancer tumor markers did not show any significant abnormalities. Epstein–Barr virus (EBV)-DNA was not detected in plasma. Pulmonary function was within the normal range. A positron emission tomography–computed tomography (PET-CT) scan revealed a metabolically active soft tissue mass in the upper lobe of the right lung with a maximum standardized uptake value (SUVmax) of approximately 19.8 and maximum dimensions of 5.3 cm by 6.7 cm. Nodules in both lungs, enlarged lymph nodes in the right hilum, enlarged lymph nodes in the mediastinum (regions 4–5), and in the left hilum were metabolically active, with SUVmax ranging from 4.3 to 21.1, indicative of metastasis (**Figure 1A**).

A fiberoptic bronchoscopic needle aspiration biopsy was conducted, extracting a neoplastic specimen from the right upper lobe and right middle bronchial opening. Subsequent histopathological examination revealed chronic inflammation of the bronchial mucosa. For further diagnosis, a CT-guided fine-needle aspiration biopsy of the right upper lung tissue, a total of six tissue samples were taken, indicating granulomatous inflammation with necrosis, suggestive of tuberculosis. However, tuberculosis (TB)-DNA detection was negative. Given the uncertainty regarding tuberculosis infection, the patient underwent a temporary course of classical quadruple antituberculosis empirical treatment for 32 days. A follow-up CT examination a month later showed no change in the lesion.

**Figure 2:**
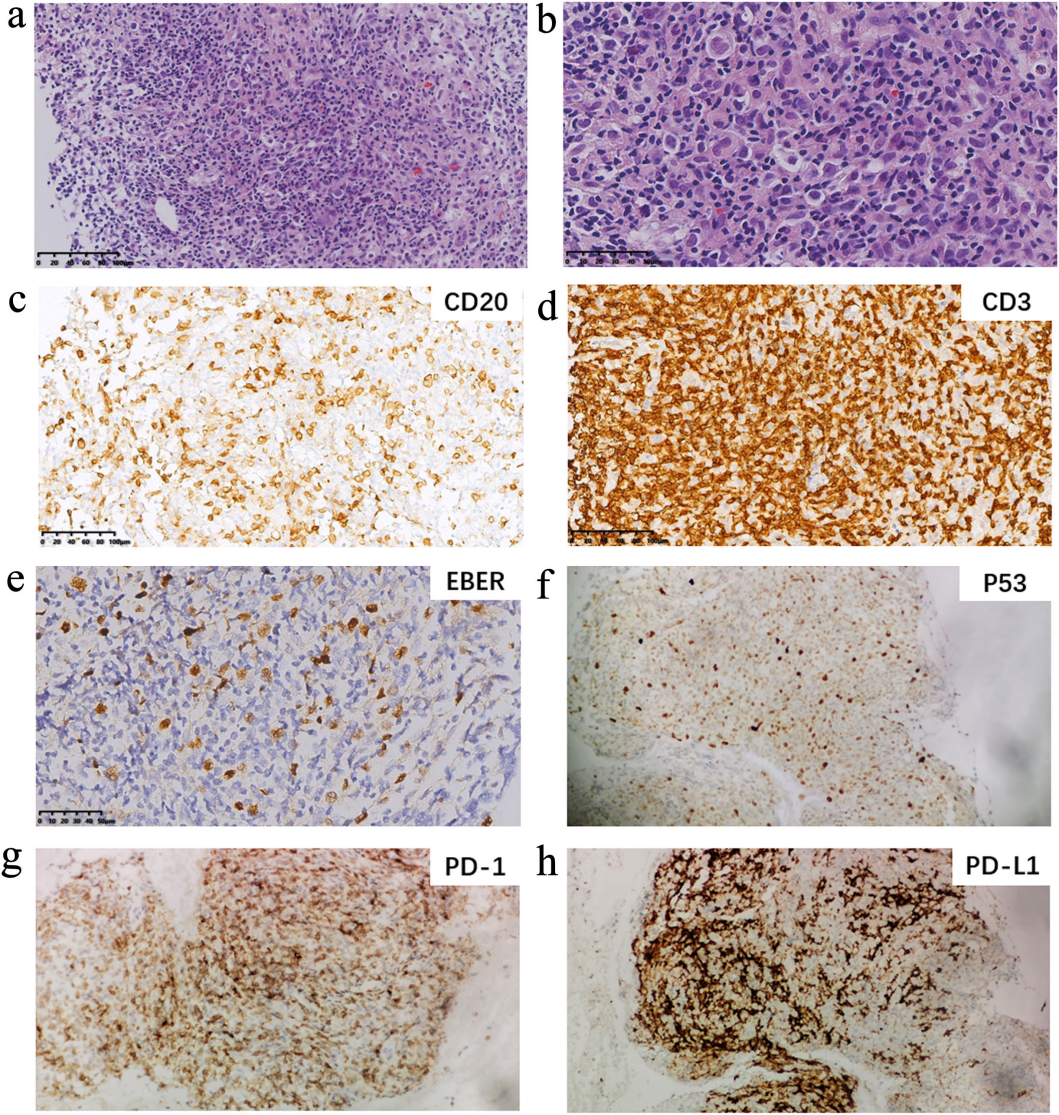
The pathological photograph in this case. **(a)** Low magnification shows typical necrotic zones. **(b)** Higher magnification of an infiltrated artery shows a predominantly large lymphoid cell infiltrate. **(c)** The large lymphoid cells stain with CD20. **(d)** the more numerous small lymphocytes stain with CD3. **(e)** Numerous EBV-positive cells are seen by ISH. The needle biopsies in this patient were positive for EBER by ISH. EBER indicates Epstein-Barr virus encoded RNA; ISH, in-situ hybridization. **(f)** Immunohistochemical stains for P53 (mouse monoclonal, clone BP53-12, Zhongshan Golden Bridge Biotechnology, Zhongshan City, Guangdong Province, China). **(g)** Immunohistochemical stains for PD-1 (mouse monoclonal, clone UMAB199, Zhongshan Golden Bridge Biotechnology, Zhongshan City, Guangdong Province, China). **(h)** Immunohistochemical stains for PD-L1 (rabbit monoclonal antibody, clone SP142, Zhongshan Golden Bridge Biotechnology, Zhongshan City, Guangdong Province, China).

A CT-guided core needle biopsy of the mass in the right upper lung was performed to evaluate the possibility of neoplastic disease, and a core tissue sample was obtained. The pathology, lymphoid immunophenotype, Epstein-Barr virus encoded RNA (EBER) ISH, and immunocytochemistry (**Figure 2**) collectively demonstrated disruption of tissue structure in one of the nodular foci in the lung tissue, which was completely replaced by lymphoid cells (the background lymphocytes were immunophenotyped as CD3 positive). Localized necrosis was observed, and the lymphocytes exhibited a mixed growth pattern. Under high magnification, the cellular infiltrate consisted of small lymphocyte-like cells, histiocytes, plasma cells, and medium- to large-sized nucleated cells resembling immunoblasts or large R-S-like cells of Hodgkin lymphoma. These larger lymphoid cells were CD20 positive and CD79a positive and partly infiltrated the blood vessels. Immunohistochemistry showed positivity for p53 (about 60%), PD-L1 (about 60%), and EBER (more than 50/HPF) in tumor cells. The background lymphocytes were PD-1 positive. The Ki-67 index was about 30% positive. Morphological correlation with CD20 on serial sections confirmed that the strong, diffuse p53 nuclear positivity was predominantly localized within the atypical CD20+ B-cell population. Next-generation sequencing revealed *IGHD* gene rearrangement. For these pathologic features, the patient was diagnosed with lymphomatoid granulomatosis, grade 3, involving the upper lobe of the right lung, multiple nodules in both lungs, and lymph nodes in the right hilar, mediastinal, and left hilar regions. Subsequently, flow cytology and lymphocyte subset analysis showed a decreased number of T cells in the bone marrow biopsy, although no abnormal tumor cells were identified.

Initially, the patient declined chemotherapy. As a result, a therapeutic regimen comprising “rituximab (an anti-CD20 monoclonal antibody [R]) plus sintilimab (a PD-1 inhibitor)” was administered. Enhanced CT scans were performed during the treatment period, revealing a partial response (PR) after two cycles (**Figure 1B**) and stable disease (SD) after four cycles (**Figure 1C**). However, a subsequent PET-CT scan after six cycles indicated an enlargement of the lesions, indicating progressive disease (PD) (**Figure 1D**).

**Figure 3:**
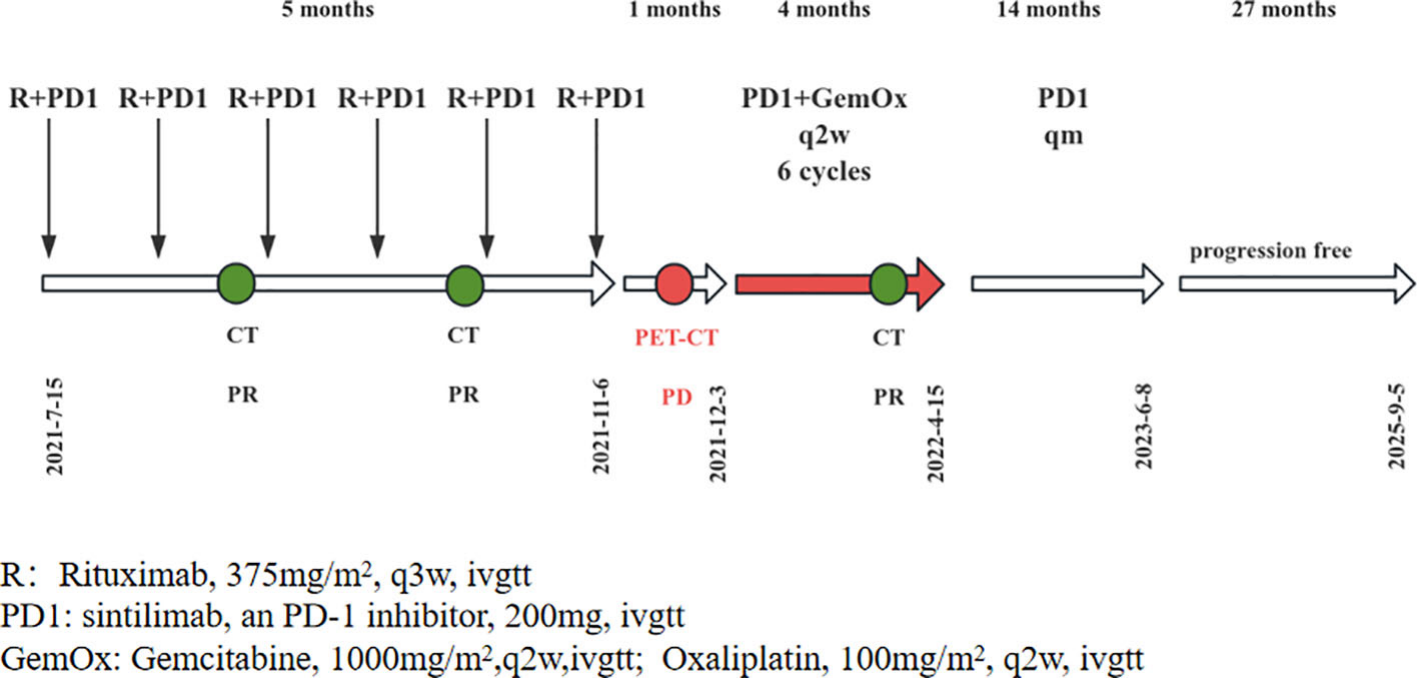
The patient’s treatment timeline and strategies.

The patient refused further rituximab treatment due to the economic constraints, leading to an adjustment in the treatment strategy to “sintilimab plus gemcitabine and oxaliplatin (GemOx)”. Enhanced CT assessments after eight (**Figure 1E**) and 12 cycles (**Figure 1F**) demonstrated a noticeable reduction in lesion size, ultimately yielding a maximum diameter of hilar lesions of less than 2.0 cm (**Figure 1G**), indicative of PR. Subsequently, the patient received sintilimab as maintenance therapy for a duration of 14 months, spanning from 15 April 2022 to 8 June 2023. At the last follow-up, the patient remained alive with an overall survival (OS) of approximately 52 months (from 8 May 2021 to 5 September 2025) and, notably, without disease progression. The patient’s treatment timeline and strategies are shown in **Figure 3**.

## Discussion

LYG is a rare clinical disease that predominantly affects middle-aged men (range: 30 to 50 years old). Laboratory results are generally normal (5), and imaging features, such as multiple lamellar and nodular shadows, are similar to those observed in infections or tumors (6). Therefore, treatment is frequently delayed because of disease misdiagnosis. Despite the use of fiberoptic bronchoscopy or CT-guided percutaneous lung aspiration biopsy, there remains a high possibility of an inconclusive diagnosis due to factors such as sampling (5).

Our patient was initially diagnosed with TB because both immunohistochemistry tests (the fiberoptic bronchoscopic needle aspiration biopsy and CT-guided fine-needle aspiration biopsy) showed granulomatous inflammation with necrosis, despite a negative TB-DNA result. Subsequently, he underwent anti-TB treatment, which proved to be ineffective.

Based on its pathologic features, PLG has been categorized as a distinct mature B-cell neoplasm in the revised World Health Organization (WHO) classification of lymphoid neoplasms in 2016 (7). The patient’s diagnosis of PLG, grade 3, was established by observing a substantial proportion of large atypical EBV+ B cells in the microscopic field (8), according to the criteria proposed by Katzenstein et al. in 2010 (9). Although high expression of p53 protein was detected by immunochemistry, next-generation sequencing (NGS), which was performed on tumor tissue (Thermo Fisher Scientific, Waltham, MA, USA, covering 38 genes including all protein-coding exons of TP53, Illumina MiniSeq platform with an average depth of coverage of 500 ×), did not show a mutation in the p53 gene. The observed strong, diffuse p53 nuclear staining by IHC alongside a wild-type NGS result may be explained by overexpression driven by nonmutational mechanisms, such as dysfunctional upstream regulation.

In 2015, the WHO stipulated that grade 3 PLG disease could be managed similarly to diffuse large B-cell lymphoma (DLBCL) (10), with a rituximab-based regimen as a first-line treatment option. Therefore, patients with PLG have generally been treated with rituximab-based strategies (11, 12), but the median survival time remains disappointingly short, at approximately 14 months. The suitability of this treatment approach for PLG remains a topic of controversy.

At present, the etiology and pathogenesis of PLG remain incompletely understood, highlighting EBV’s capacity to bind to the CD21 receptor on the surface of B cells, leading to monoclonal proliferation of B lymphocytes, particularly in immunodeficient states (3). In this case, both bone marrow flow cytometry and lymphocyte subset analysis showed a reduced number of T cells, potentially facilitating immune escape by abnormal B cells and their eventual transformation into malignant tumors (13).

The engagement of PD-1 on T cells by PD-L1/PD-L2 on tumor cells leads to suppression of T-cell effector functions and proliferation, facilitating immune evasion. Therapeutic blockade of the PD-1/PD-L1 axis aims to reverse this inhibition and restore antitumor immunity (14). One abstract reported a very high overall response rate of 75% (three of four) in LYG patients with nivolumab (480 mg, iv, q4w) monotherapy (15). Unfortunately, two patients experienced disease progression, indicating that the efficacy of monotherapy may not be durable. As a result, a PD-1 inhibitor-based combination therapy should be considered. Immunohistochemistry findings indicated high PD-1 and PD-L1 expression in our patient. Based on these findings, a PD-1 inhibitor-based regimen was initiated. To date, the patient has undergone 14 months of treatment and remains alive, with an overall survival of 52 months, exceeding the reported median survival time.

In conclusion, PLG is an aggressive disease. PD-L1 expression warrants evaluation as a potential biomarker to guide PD-1 inhibitor therapy in PLG, a hypothesis that requires validation in larger cohorts.

## Data Availability

The datasets presented in this article are not readily available because Our manuscript did not include any datasets. Requests to access the datasets should be directed to JM fccmaj2@zzu.edu.cn.
